# Effect of curcumin combined with vedolizumab on disease activity in patients with moderate to severe ulcerative colitis and its correlation with inflammatory and immune factors

**DOI:** 10.3389/fmed.2026.1789083

**Published:** 2026-04-02

**Authors:** Ye Zhao, Ming Bao, Wencui Niu

**Affiliations:** 1 Gastroenterology Department, Beijing Hospital of Integrated Traditional Chinese and Western Medicine, Beijing, China; 2 Cadre Diagnosis and Treatment Department, PLA General Hospital, Beijing, China; 3 Nutrition Department, Beijing Hospital of Integrated Traditional Chinese and Western Medicine, Beijing, China

**Keywords:** curcumin, disease activity index, immune factor, ulcerative colitis, vedolizumab

## Abstract

**Objective:**

To investigate the clinical efficacy and immunomodulatory effects of curcumin (Cur) combined with vedolizumab (VDZ) in patients with moderate-to-severe ulcerative colitis (UC).

**Methods:**

A prospective, multicenter, randomized controlled double-blind study was conducted. Patients with moderate-to-severe UC admitted between March 2021 and March 2024 were enrolled and randomly assigned to either the VDZ monotherapy group (76 cases) or the VDZ combined with Cur combination group (83 cases). The treatment period was 26 weeks. The primary outcome was the clinical remission rate at 26 weeks. Secondary outcomes included the remission rate at 14 weeks, the disease activity index (DAI), inflammatory and immune markers, and safety.

**Results:**

The combination group showed significantly higher clinical remission rates at both 26 and 14 weeks compared to the VDZ group (*P* < 0.05). DAI scores decreased significantly over time in both groups (*P* < 0.05), with a greater reduction observed in the combination group at both 14 and 26 weeks (*P* < 0.05). The combination group demonstrated more significant improvements in inflammatory and immune markers: at 26 weeks, CRP, Th17/Treg ratio, and fecal calprotectin levels were lower than those in the VDZ group (*P* < 0.05), while IL-10 levels were higher at 14 weeks (*P* < 0.05). No statistically significant difference in the incidence of adverse reactions was observed between the two groups (*P* > 0.05), indicating comparable safety profiles. In the combination group, DAI exhibited a stronger positive correlation with Th17/Treg ratio, CRP, and fecal calprotectin, and a more pronounced negative correlation with IL-10 (*P* < 0.05). Subgroup analysis showed that both the VDZ group and the combination group had higher benefits in moderate UC compared to severe UC.

**Conclusion:**

Curcumin as an combination group to VDZ significantly improves clinical remission rates, alleviates inflammatory and immune imbalances in patients with moderate-to-severe UC, without increasing safety risks, highlighting its important clinical translational value. This study provides new evidence for combination treatment strategies in UC.

## Introduction

Ulcerative colitis (UC) is an immune-mediated chronic non-specific inflammatory bowel disease (IBD) characterized by progressive damage to the intestinal mucosa (1). The global incidence of UC is rising at a rate of 4%−15% annually, drawing widespread public health concern (2). Moderate to severe ulcerative colitis accounts for approximately one-fourth of all UC cases. Clinically, it often presents as persistent or recurrent diarrhea, bloody mucopurulent stools, abdominal pain, and other symptoms. Some patients also experience extraintestinal manifestations and face a higher risk of cancer development, with the disease often being protracted and difficult to resolve (3). Studies have shown that the mortality rates among patients with moderate to severe UC reach 0.84% at 3 months and 1.01% at 12 months, posing a significant threat to patient health (4). Currently, UC has no cure, and the core objectives of its treatment are to induce and maintain clinical remission, ultimately improving patients' quality of life (5). Achieving these goals depends heavily on the severity of the disease. In particular, moderate to severe UC presents a major clinical challenge due to its complex nature, high relapse rates, and poor prognosis. Additionally, the disease's significant heterogeneity and the immunogenicity of medications limit therapeutic efficacy, further complicating management (6).

Given this clinical challenge, exploring new treatment strategies to optimize existing regimens has become particularly important. Vedolizumab (VDZ), a gut-selective integrin inhibitor, works by specifically blocking the binding of α4/β7 integrin to mucosal addressin cell adhesion molecule-1 (MAdCAM-1), precisely inhibiting the migration of lymphocytes to intestinal inflammatory sites. This mechanism offers unique efficacy and safety profiles (7). However, the clinical efficacy of single-drug therapy is often limited, and the role of traditional Chinese medicine (TCM) in prevention and treatment has gradually gained attention (8). Curcumin (Cur), a natural polyphenol with potent anti-inflammatory and antioxidant activities, exhibits effects such as reducing inflammation and improving microcirculation (9). It can help eliminate blood stasis in UC on top of the immunomodulatory action of VDZ, thereby preventing stasis from obstructing angiogenesis and ulcer healing, which may enhance the therapeutic effect of VDZ (10). Research indicates that Cur can modulate intestinal epithelial permeability by inhibiting key inflammatory pathways, such as NF-κB, suppress apoptosis of intestinal epithelial cells, and subsequently alleviate colonic inflammation (11). After treatment with curcumin, significant improvements were observed in long-term survival rates and body weight in rats (12). Therefore, combining the precise immunomodulatory action of VDZ with the multi-target anti-inflammatory advantages of Cur holds promise for achieving synergistic effects, offering a potentially promising new approach to overcome the treatment bottleneck in moderate-to-severe UC.

Disease activity index (DAI) is a key comprehensive measure for assessing the severity of ulcerative colitis and treatment response, and it can serve as the primary outcome for evaluating the clinical efficacy of the aforementioned combination therapy (2). However, the underlying mechanisms of the therapeutic effects require further elucidation through their correlation with specific inflammatory and immune markers that reflect the core pathophysiological processes of UC (13). In UC, cellular immunity and inflammatory factors directly contribute to tissue damage and mucosal inflammation, thereby triggering immune responses under specific conditions (14). Numerous inflammatory mediators are involved in the pathogenesis of UC (13). C-reactive protein (CRP), a key indicator of systemic inflammatory burden, serves as an important inflammatory factor in both screening and monitoring stages of UC (15). Fecal calprotectin demonstrates a strong correlation with clinical response, endoscopic parameters, and mucosal healing in UC (16). Additionally, studies have shown that dysregulated immune responses mediated by helper T cell 17 (Th17)/regulatory T cell (Treg) are hallmark features of UC pathogenesis (17). IL-10, as an anti-inflammatory cytokine, is a critical component in maintaining intestinal immune homeostasis (18). By inhibiting pro-inflammatory factors, it indirectly helps preserve the integrity of the intestinal epithelial barrier and is also a significant indicator influencing UC (18). Clinically, research on the efficacy of VDZ and Cur in alleviating moderate-to-severe UC remains insufficient. Based on this, the present study aims to evaluate the effects of VDZ and Cur on the DAI in patients with moderate-to-severe UC and its correlation with key inflammatory biomarkers, thereby providing new insights into the potential role of VDZ and Cur in the treatment of moderate-to-severe UC.

## Material and methods

### Study subjects

This prospective, multicenter randomized controlled study was conducted at Beijing Hospital of Integrated Traditional Chinese and Western Medicine and the Chinese PLA General Hospital. Using DAI as the study endpoint, the sample size was estimated with α = 0.01 and 1–β = 0.90. Based on preliminary experimental results, the post-treatment DAI values for the VDZ group and the combination group were determined as μ_1_ = 142.7 and μ_2_ = 117.8, with standard deviations of σ_1_ = 30.6 and σ_2_ = 35.2, respectively. PASS 15.0 (NCSS, LLC, Kaysville, Utah, USA) software calculated the required sample size as *N*_1_ = *N*_2_ = 62, resulting in a total sample size of 124. Accounting for a potential attrition rate of 20%, the required sample size for inclusion was adjusted to *N*_1_ = *N*_2_ = 78, totaling 156 participants.

**Figure 1:**
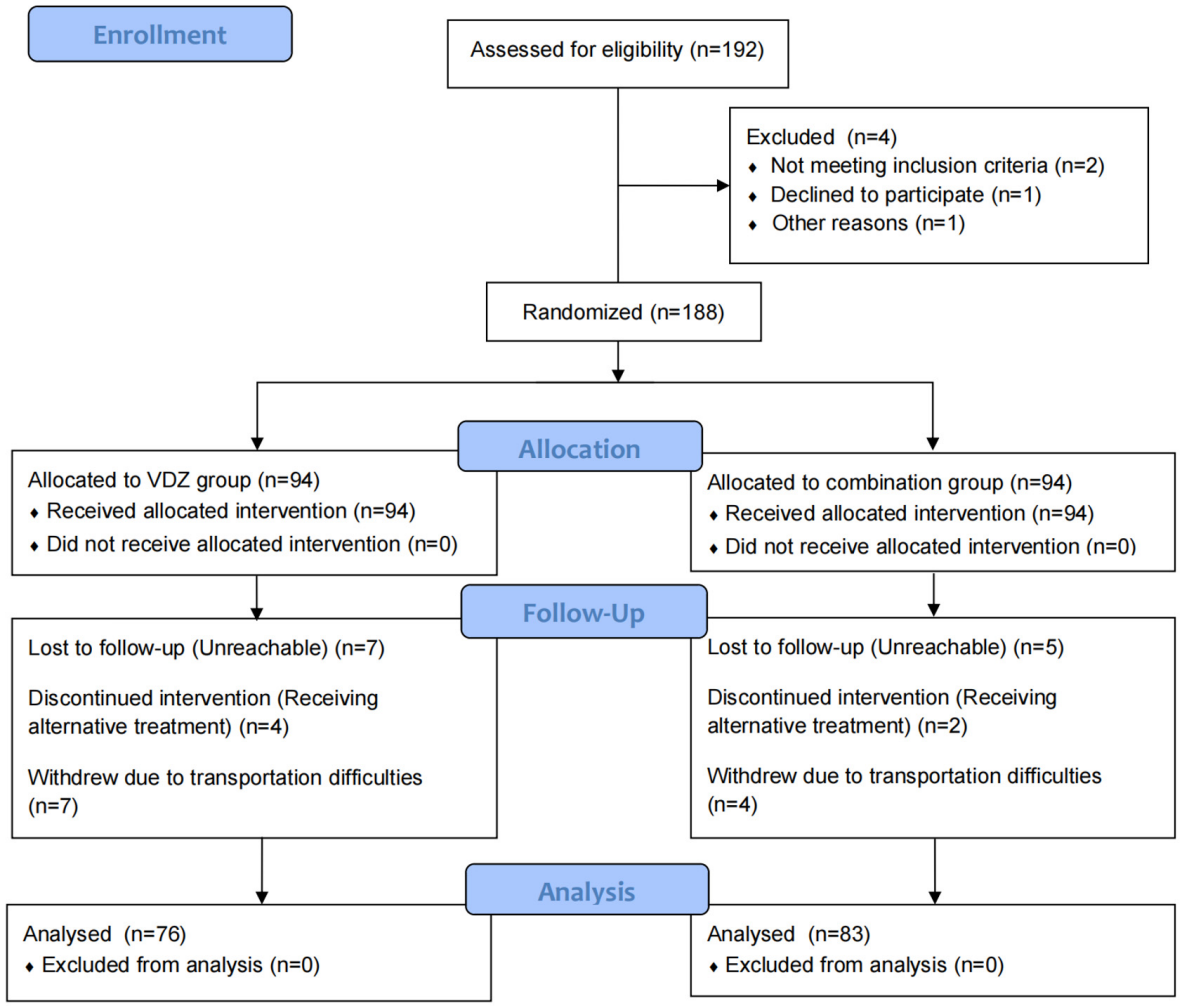
Patient enrollment flowchart.

A total of 188 patients with moderate to severe UC were prospectively enrolled from Beijing Hospital of Integrated Traditional Chinese and Western Medicine and the Chinese PLA General Hospital between March 2021 and March 2024. Using a random number table, all participants were divided into two groups: the VDZ group and the combination group, with 94 patients in each group. The VDZ group received VDZ treatment, while the combination group received VDZ treatment combined with Cur therapy. The treatment duration was 26 weeks. During the study, 11 patients withdrew due to self-reported transportation inconvenience, 12 were lost to follow-up, and six were excluded because their treatment duration was insufficient (< 26 weeks) for personal reasons. A total of 159 patients were eventually included, which slightly exceeded the planned sample size because additional eligible patients agreed to participate and were randomized before the recruitment window closed (Figure 1). Among them, 76 patients in the VDZ group completed the full treatment, and 83 patients in the combination group received the complete treatment. Prior to the study initiation, ethical approval was obtained from the review boards of Beijing Hospital of Integrated Traditional Chinese and Western Medicine and the Chinese PLA General Hospital (Ethics Approval No.: ZXYEC-KT-2020-01-P01). The study has been approved by the Chinese Clinical Trial Registry (Clinical Registration Number: ChiCTR2500119833). The study was conducted in accordance with the principles of the Declaration of Helsinki. Written informed consent was obtained from all participants before randomization.

Inclusion criteria: (1) diagnosis of moderate-to-severe UC according to the adult ulcerative colitis diagnostic guidelines, based on clinical, endoscopic, and histological examinations (19); (2) age ≥18 years; (3) no history of intestinal surgery within the past 6 months; (4) for female patients, provision of effective contraception measures and a negative pregnancy test; and (5) patients provided informed consent. Exclusion criteria: (1) history of VDZ use or allergy to VDZ or its excipients; (2) presence of severe hepatic or renal dysfunction, or insufficiency of other vital organs; and (3) current or recent use of other experimental therapeutic agents. Criteria for exclusion and withdrawal: (1) patient requests to withdraw from the study due to personal reasons; (2) patient is lost to follow-up; and (3) failure to complete the full course of treatment.

### Drug treatment methods

VDZ group: received intravenous vedolizumab (Cilag AG, Schaffhausen, Switzerland, Approval No. S2017001, 300 mg/vial) at a dose of 300 mg per administration. The dosing regimen involved reconstituting vedolizumab with sterile water for injection, diluting it in 250 mL of sterile 0.9% sodium chloride solution, and administering it via continuous intravenous infusion over 30 min. After the infusion was completed, the line was flushed with 30 mL of sterile 0.9% sodium chloride solution. Infusions were administered at weeks 0, 2, and 6, followed by the same dose every 8 weeks thereafter, for a total duration of 26 weeks.

Combination group: in addition to the medication regimen of the VDZ group, patients in this group also received curcumin preparation [Jianghuangsu Zhitongluo Soft Capsules (Zhi Keping), manufactured by Shineway Pharmaceutical Group Co., Ltd., Wan Chai, Hong Kong, National Medicine Approval No. Z20040032, containing 50 mg of curcumin compounds per capsule] at a dose of 100 mg per administration, three times daily, for a duration of 26 weeks.

During the study period, patients were permitted to continue using 5-aminosalicylates and immunomodulators that had been administered at stable doses prior to enrollment, and were required to maintain the same dosage throughout the study. For patients using corticosteroids at the time of enrollment, a standardized tapering regimen was established, and dosage adjustments were documented at each follow-up visit. Initiation of other biologics or JAK inhibitors during the study period was prohibited.

### Randomization and blinding

A block randomization method was employed in this study, with random sequences generated by an independent statistician not involved in clinical assessments using SAS 9.4 (SAS Institute Inc., Cary, North Carolina, USA) software (block size of 4). Allocation results were sealed in sequentially numbered, opaque, sealed envelopes and maintained by a dedicated research nurse who was not involved in patient recruitment or evaluation. Upon enrollment, the research nurse opened the envelopes in sequential order and dispensed medications according to the allocation results.

To maintain blinding, a double-dummy technique was adopted. Patients in the VDZ monotherapy group received oral placebo capsules identical in appearance, size, color, and odor to curcumin (mainly composed of starch, provided by Shijiazhuang Pharmaceutical Group, Shijiazhuang, Hebei, China), administered three times daily, two capsules each time, for 26 weeks, in addition to VDZ intravenous infusion. Patients in the combination therapy group received oral curcumin capsules (National Medicine Approval No. Z20040032) at the same dosage alongside VDZ infusion. All patients underwent the same frequency and mode of VDZ intravenous infusions and oral capsule administration, ensuring that patients, clinicians, outcome assessors, and statisticians remained blinded to group allocation.

Outcome assessment was conducted by trained gastroenterologists who were not involved in patient treatment. All data entry and statistical analyses were performed using coded group assignments by independent statisticians prior to unblinding. Unblinding was not performed until data analysis was completed and preliminary conclusions were formed.

### Inflammatory marker detection methods

Serum C-reactive protein levels were measured using a double-antibody sandwich enzyme-linked immunosorbent assay, with kits purchased from R&D Systems China Co., Ltd., Changning District, Shanghai, China (Catalog No.: DCRP00). All procedures were strictly performed according to the manufacturer's instructions, with each sample tested in duplicate and the mean value used for analysis. Serum IL-10 levels and fecal calprotectin levels were detected using ELISA kits purchased from Shanghai Senruidiao Biotechnology Co., Ltd., Pudong New District, Shanghai, China (IL-10 Catalog No.: SRL-IL10-HU; calprotectin Catalog No.: SRL-CAL-HU). Pre-experiments were conducted for all samples to determine the optimal dilution factor prior to formal testing.

### Th17/Treg cell detection methods

Flow cytometry was employed to detect the proportions of Th17 and Treg cells in peripheral blood. The specific procedures were as follows: 2 mL of venous blood was collected into EDTA anticoagulant tubes and processed within 4 h. A volume of 100 μL of whole blood was added to 5 mL flow cytometry tubes, followed by staining with corresponding surface antibodies: CD4-FITC (BD Biosciences, Shanghai, China, Catalog No. 555346) and CD25-PE (BD Biosciences, Catalog No. 555432), incubated at 4 °C in the dark for 30 min. Then, 2 mL of red blood cell lysis buffer (BD Biosciences, Catalog No. 555899) was added and incubated at room temperature in the dark for 10 min. After centrifugation and supernatant removal, 1 mL of fixation/permeabilization buffer (eBioscience, San Diego, California, USA, Catalog No. 00-5523-00) was added and incubated at 4 °C in the dark for 45 min. Following washing, intracellular antibodies were added: FoxP3-APC (eBioscience, Catalog No. 17-4776-42) for Treg detection; alternatively, cells were stimulated with cell stimulation cocktail (PMA 50 ng/mL + ionomycin 1 μg/mL) and monensin (BD Biosciences, Catalog No. 554724) for 4–6 h of culture, followed by surface staining, fixation/permeabilization, and subsequent intracellular staining with IL-17A-PE (eBioscience, Catalog No. 12-7179-42). Detection was performed using a BD FACSCanto II flow cytometer, with at least 50,000 lymphocytes acquired per tube. Data analysis was conducted using FlowJo V10 software (BD Biosciences).

### Efficacy evaluation

According to the study by scholars such as Binsaleh et al. (2), the DAI was calculated for each patient. The formula for DAI is: DAI = 13 × (number of bowel movements) + 60 × (amount of blood in stool) + 0.5 × (erythrocyte sedimentation rate, ESR)-4 × (hemoglobin, Hb)-15 × (albumin) + 200. Index values below 150 correspond to mild disease, those between 150 and 220 correspond to moderate disease, and values above 220 correspond to severe disease. Here, the number of bowel movements reflects diarrhea frequency as an indicator for assessing disease severity; blood in stool is an important marker of mucosal inflammation and ulceration; ESR is a marker of systemic inflammation; Hb reflects the impact of chronic inflammation and potential blood loss; serum albumin is a marker of nutritional status and disease severity; and the constant factor (200) is used for scale normalization. Patients in both groups were assessed at weeks 0, 14, and 26, with remission rates calculated for weeks 14 and 26. The primary efficacy outcome was the remission rate at 26 weeks, which evaluated long-term efficacy. Therapeutic efficacy was categorized based on the reduction rate of the DAI from baseline: a reduction of < 30 points was defined as ineffective; a reduction of 30–70 points as effective; and a reduction of ≥70 points as remission (2).

Mayo endoscopic subscore (Mayo ES) classification consists of four grades: Grade 0 (normal or inactive disease); Grade 1 (mild disease, characterized by erythema, decreased vascular pattern, and mild friability); Grade 2 (moderate disease, characterized by marked erythema, absent vascular pattern, friability, and erosions); Grade 3 (severe disease, characterized by spontaneous bleeding and ulceration) (13).

### Histopathological assessment

Before and after treatment, the Robarts Histopathology Index (RHI) was assessed via colonoscopy. The RHI primarily evaluates four histological features: lamina propria neutrophils, epithelial neutrophils, chronic inflammatory cell infiltration, erosion or ulceration, with a total maximum score of 33 points. A higher score indicates more severe disease (20).

### Safety evaluation

The Common Terminology Criteria for Adverse Events (CTCAE) (21) were used to describe the types and severity of adverse reactions occurring in both groups of patients from week 0 to 26. The CTCAE includes a severity grading scale; therefore, any adverse event (AE) reported was annotated with its corresponding severity grade (Table 1). The incidence of adverse drug reactions (ADR), odds ratio (OR), and relative risk (RR) were employed to assess the safety profiles of Cur and VDZ.

**Table 1:** CTCAE grades response choices/associated scores.

CTCAE grade	Description of grading criteria
0	No adverse event (or within normal limits)
1	Mild; asymptomatic or mild symptoms; clinical or observations only; intervention not indicated
2	Moderate; minimal, local, or non-invasive intervention indicated; limiting age-appropriate instrumental activities of daily living (ADL)
3	Severe or medically significant but not immediately life-threatening; hospitalization or prolongation of hospitalization indicated; disabling; limiting self-care ADL
4	Life-threatening consequences; urgent intervention indicated
5	Death related to adverse event

### Sample collection

At baseline (before treatment), week 14, and week 26 of treatment, 10 mL of blood was drawn from the antecubital vein. The blood was then aliquoted into tubes and allowed to clot. The samples were centrifuged at 4,500 g for 10 min using a Hettich EBA 20 centrifuge. The resulting serum samples were stored at −80 °C for subsequent measurement of specific cytokine levels. Fecal samples were weighed, homogenized in saline, and centrifuged. The clarified supernatant was collected for the analysis of fecal calprotectin. Flow cytometry was used to determine the percentages of Th17 and Treg cells, and the Th17/Treg ratio was calculated. Serum levels of IL-10 and CRP, as well as fecal calprotectin levels, were measured using commercially available enzyme-linked immunosorbent assay (ELISA) kits according to the manufacturers' instructions. The CRP ELISA kit was supplied by R&D Systems China Co., Ltd., and the remaining kits were supplied by Shanghai Senruidiao Co., Ltd., China.

### Statistical analysis

Data analysis and graph creation were performed using SPSS 27.0 software (IBM Corporation, Armonk, NY, United States) and Prism version 10.0 (GraphPad, San Diego, CA, United States). Normality was assessed using the Shapiro-Wilk test. Quantitative data with a normal distribution are expressed as mean ± standard deviation (*x* ± *s*) and compared between two groups using the independent samples *t*-test. Quantitative data with a non-normal distribution are expressed as median (first quartile, third quartile) [M (P_25_, P_75_)] and analyzed using the Mann-Whitney *U*-test for comparisons between two groups. For normally distributed quantitative data across different time points, repeated measures ANOVA was used. For non-normally distributed quantitative data across different time points, generalized estimating equations were applied. Count data are expressed as number of cases (percentage) [*n* (%)] and analyzed using the χ^2^-test. Ranked variables are expressed as number of cases (rate) [*n* (%)] and analyzed using the rank-sum test. Correlations were determined using the Pearson correlation coefficient test. All *P*-values were two-tailed, and a *P* < 0.05 was considered statistically significant.

## Results

### Comparison of baseline characteristics between the two groups

There were no statistically significant differences between the two groups in terms of age, gender, body weight (BW), Montreal classification, severity grade, disease duration, type of onset, alcohol habits, smoking habits, and concomitant medications (*P* > 0.05; Table 2).

**Table 2:** Comparison of baseline characteristics between the two groups.

Characteristics	VDZ group (*n* = 76)	Combination group (*n* = 83)	*t*/*Z*/χ^2^	*P*
Age, years (*x* ± *s*)	51.37 ± 11.60	50.76 ± 12.44	0.319	0.750
Gender [*n* (%)]				
Male	41 (53.95)	44 (53.01)	0.014	0.906
Female	35 (46.05)	39 (46.99)		
BW, kg [M (P_25_, P_75_)]	55.68 (45.85, 67.15)	58.05 (50.95, 67.85)	−1.307	0.191
Montreal classification [*n* (%)]				
E1	37 (48.68)	35 (42.17)	1.301	0.522
E2	32 (42.11)	36 (43.37)		
E3	7 (9.21)	12 (14.46)		
Severity grade [*n* (%)]				
Moderate	26 (34.67)	24 (28.92)	0.516	0.473
Severe	50 (65.79)	59 (71.08)		
Disease duration, months [M (P_25_, P_75_)]	21.00 (14.00, 31.00)	18.00 (11.00, 28.00)	−1.623	0.105
Type of onset [*n* (%)]				
First episode	54 (71.05)	65 (78.31)	1.111	0.292
Relapse	22 (28.95)	18 (21.69)		
Alcohol habits [*n* (%)]				
Yes	12 (15.79)	17 (20.48)	0.586	0.444
No	64 (84.21)	66 (79.52)		
Smoking habits [*n* (%)]				
Yes	38 (50.00)	44 (53.01)	0.144	0.704
No	38 (50.00)	39 (46.99)		
Concomitant medications [*n* (%)]				
5-aminosalicylates	52 (68.42)	59 (71.08)	0.134	0.714
Corticosteroids	28 (36.84)	31 (37.35)	0.004	0.947
Immunomodulators	19 (25.00)	21 (25.30)	0.002	0.965
Prior biologic exposure	15 (19.74)	18 (21.69)	0.092	0.762

### Improvement in hematological parameters

The white blood cell (WBC) counts in both groups showed significant improvement at week 26 of treatment compared to week 0 (*P* < 0.05). The ESR in the combination group was significantly better at week 14 of treatment compared to week 0 (*P* < 0.05), and also superior to the VDZ group (*P* < 0.05). The red blood cell (RBC) counts in both groups were significantly better at weeks 14 and 26 of treatment compared to week 0 (*P* < 0.05). The PLT in the combination group was significantly better at week 26 of treatment compared to week 0 (*P* < 0.05), and also superior to the VDZ group (*P* < 0.05). The ALC in both groups was significantly better at weeks 14 and 26 of treatment compared to week 0 (*P* < 0.05). Additionally, the ALC in the combination group was significantly better at week 26 of treatment compared to week 0 (*P* < 0.05), and also superior to the VDZ group (*P* < 0.05; Table 3).

**Table 3:** Generalized estimating equations model for the improvement of routine blood parameters between the two groups.

Characteristics	Time	VDZ group (*n* = 76)	Combination group (*n* = 83)	Time effect	HR (95% CI)	Interaction effect	HR (95% CI)
				*P*		*P*	
Hb, g/L	0 week	40.63 (11.63, 82.29)	50.80 (11.61, 73.74)	–	–	–	–
	14 weeks	42.97 (21.66, 63.14)	44.37 (17.54, 66.56)	0.282	0.031 (0.006–1.718)	0.489	2.166 (0.009–5.231)
	26 weeks	42.34 (23.87, 61.72)	45.03 (25.39, 60.15)	0.095	0.006 (0.001–2.462)	0.439	1.341 (0.009–2.107)
Group effect	*P*	–	0.758				
HR (95% CI)		–	0.172 (0.023–1.261)				
WBC count, 10^9^/L	0 week	6.40 (5.70, 7.20)	6.48 (5.87, 7.27)	–	–	–	–
	14 weeks	6.21 (5.56, 6.76)	6.23 (5.57, 7.29)	0.122	0.788 (0.582–1.066)	0.973	0.993 (0.641–1.538)
	26 weeks	5.78 (5.04, 6.37)	5.77 (5.10, 6.70)	0.001	0.565 (0.409–0.781)	0.923	1.025 (0.619–1.696)
Group effect	*P*	–	0.319				
HR (95% CI)		–	1.182 (0.851–1.641)				
ESR, mm/h	0 week	23.22 (21.53, 25.39)	24.46 (21.87, 26.27)	–	–	–	–
	14 weeks	23.59 (20.93, 26.87)	23.27 (21.01, 25.23)	0.835	1.077 (0.534–2.173)	0.015	0.293 (0.110–0.785)
	26 weeks	24.70 (20.06, 28.05)	24.33 (20.56, 27.01)	0.454	1.696 (0.425–6.764)	0.374	0.420 (0.062–2.842)
Group effect	*P*	–	0.277				
HR (95% CI)		–	1.790 (0.627–5.113)				
ALB, g/L	0 week	32.60 (26.71, 36.21)	32.32 (27.67, 35.73)	–	–	–	–
	14 weeks	31.69 (27.58, 34.51)	32.40 (28.32, 35.81)	0.421	0.559 (0.136–2.300)	0.299	2.567 (0.432–15.264)
	26 weeks	31.59 (28.46, 34.73)	32.75 (29.07, 34.86)	0.704	0.771 (0.202–2.944)	0.468	2.070 (0.290–14.783)
Group effect	*P*	–	0.955				
HR (95% CI)		–	0.941 (0.115–7.677)				
RBC count, 10^9^/L	0 week	4.05 (3.42, 4.84)	4.11 (3.48, 4.76)	–	–	–	–
	14 weeks	4.48 (3.90, 5.31)	4.75 (4.19, 5.38)	0.005	1.478 (1.126–1.941)	0.085	1.404 (0.954–2.067)
	26 weeks	5.21 (4.70, 5.99)	5.24 (4.73, 5.82)	< 0.001	3.118 (2.379–4.086)	0.315	1.234 (0.819–1.858)
Group effect	*P*	–	0.588				
HR (95% CI)		–	0.924 (0.695–1.230)				
PLT, 10^9^/L	0 week	241.10 (89.25, 380.76)	199.19 (77.06, 317.91)	–	–	–	–
	14 weeks	265.02 (179.93, 351.33)	285.37 (197.90, 365.87)	0.310	2.087 (2.060–2.114)	0.106	0.569 (0.370–0.876)
	26 weeks	248.31 (191.18, 364.11)	340.61 (236.32, 424.50)	0.116	0.635 (0.010–3.279)	0.012	0.231 (0.209–1.980)
Group effect	*P*	–	0.295				
HR (95% CI)		–	1.477 (0.803–2.719)				
ALC, 10^9^/L	0 week	1.54 (1.29, 1.85)	1.65 (1.29, 1.89)	–	–	–	–
	14 weeks	1.73 (1.27, 2.08)	1.80 (1.40, 2.17)	0.037	1.193 (1.011–1.407)	0.831	1.025 (0.818–1.284)
	26 weeks	2.18 (1.74, 2.60)	2.43 (2.19, 2.83)	< 0.001	1.876 (1.574–2.235)	0.020	1.331 (1.046–1.695)
Group effect	*P*	–	0.328				
HR (95% CI)		–	1.067 (0.937–1.215)				

### Improvement in immunological parameters

The group-by-time interactions for CRP, Th17/Treg ratio, and calprotectin were all statistically significant (*P* < 0.05). In the combination group, the expression levels of all three factors were significantly lower than those in the VDZ group at week 26 (*P* < 0.05). For both groups, all three indicators were significantly lower at weeks 14 and 26 compared to week 0 (*P* < 0.05). In the combination group, IL-10 was significantly higher than in the VDZ group at week 14 (*P* < 0.05), and its levels were significantly higher at both weeks 14 and 26 compared to week 0 (*P* < 0.05; Table 4).

**Table 4:** Repeated-measures analysis of variance for improvement in immunological indicators between the two groups (*x* ± *s*).

Characteristics	0 week	14 weeks	26 weeks	Mauchly *W*	*P*	*F*	*P*	Partial eta squared
CRP, pg/mL								
VDZ group (*n* = 76)	154.09 ± 11.34	83.91 ± 9.10^*^	52.48 ± 10.33^*#^	0.975	0.142	6.600	0.002	0.040
Combination group (*n* = 83)	155.90 ± 10.81	84.09 ± 8.62^*^	46.51 ± 10.78^*#^					
*t*	−1.030	−0.131	3.554					
*P*	0.304	0.896	0.001					
95% CI	−5.281 to 1.668	−2.960 to 2.592	2.649–9.276					
Th17/Treg								
VDZ group (*n* = 76)	2.52 ± 0.50	1.74 ± 0.57^*^	1.45 ± 0.57^*#^	1.000	0.987	12.477	< 0.001	0.074
Combination group (*n* = 83)	2.65 ± 0.53	1.84 ± 0.56^*^	1.06 ± 0.43^*#^					
*t*	−1.556	−1.096	4.860					
*P*	0.122	0.275	< 0.001					
95% CI	−0.288 to 0.034	−0.274 to 0.079	0.231–0.548					
IL-10, pg/mL								
VDZ group (*n* = 76)	172.56 ± 19.56	173.04 ± 14.43	181.51 ± 18.72^*#^	0.978	0.172	2.274	0.105	0.014
Combination group (*n* = 83)	170.48 ± 18.46	177.76 ± 13.39^*^	187.21 ± 20.97^*#^					
*t*	0.689	−2.140	−1.799					
*P*	0.492	0.034	0.074					
95% CI	−3.877 to 8.034	−9.078 to −0.364	−11.941 to 0.556					
Calprotectin, ng/mL								
VDZ group (*n* = 76)	28.84 ± 3.80	25.38 ± 4.17^*^	20.32 ± 4.79^*#^	0.997	0.783	6.867	0.001	0.042
Combination group (*n* = 83)	29.07 ± 4.05	24.74 ± 4.73^*^	17.20 ± 4.59^*#^					
*t*	−0.376	0.898	4.188					
*P*	0.707	0.370	< 0.001					
95% CI	−1.468 to 0.998	−0.765 to 2.041	1.648–4.590					

^*^P < 0.05 compared with the value at 0 week.

^#^*P* < 0.05 compared with the value at 14 weeks.

### Symptom improvement and efficacy

There were statistically significant time effects in abdominal pain, diarrhea, hematochezia, bowel movement (BM), and volume of hematochezia between the two groups of patients (*P* < 0.05). The interaction effect for diarrhea symptoms showed a significant difference between the two groups at week 14 (*P* < 0.05). The interaction effect for hematochezia symptoms showed significant differences between the two groups at weeks 14 and 26 (*P* < 0.05; Table 5).

**Table 5:** Comparison of symptom remission rates between the two groups.

Characteristics	Time	VDZ group (*n* = 76)	Combination group (*n* = 83)	Time effect	HR (95% CI)	Interaction effect	HR (95% CI)
				*P*		*P*	
Abdominal pain, times/day	0 week	5.50 (4.00, 7.00)	5.00 (4.00, 7.00)	–	–	–	–
	14 weeks	4.00 (3.00, 5.00)	3.00 (2.00, 4.00)	< 0.001	0.158 (0.115–0.218)	0.295	0.814 (0.553–1.197)
	26 weeks	2.00 (2.00, 3.00)	2.00 (1.00, 3.00)	< 0.001	0.037 (0.026–0.053)	0.269	0.767 (0.480–1.227)
Group effect	*P*	–	0.218				
HR (95% CI)		–	0.680 (0.369–1.256)				
Diarrhea, times/day	0 week	3.00 (1.25, 4.00)	3.00 (2.00, 4.00)	–	–	–	–
	14 weeks	2.00 (1.00, 3.00)	2.00 (0.00, 2.00)	< 0.001	0.349 (0.282–0.432)	0.017	0.708 (0.533–0.941)
	26 weeks	1.00 (0.00, 2.00)	1.00 (0.00, 2.00)	< 0.001	0.214 (0.167–0.276)	0.158	0.784 (0.559–1.099)
Group effect	*P*	–	0.805				
HR (95% CI)		–	1.067 (0.638–1.784)				
Hematochezia, times/day	0 week	3.00 (2.00, 5.00)	3.00 (2.00, 4.00)	–	–	–	–
	14 weeks	3.00 (2.00, 4.00)	2.00 (2.00, 3.00)	< 0.001	0.692 (0.607–0.788)	< 0.001	0.637 (0.519–0.782)
	26 weeks	3.00 (2.00, 4.00)	2.00 (2.00, 3.00)	< 0.001	0.599 (0.514–0.697)	< 0.001	0.572 (0.448–0.730)
Group effect	*P*	–	0.918				
HR (95% CI)		–	1.023 (0.664–1.576)				
BM, times/day	0 week	5.00 (4.00, 6.00)	5.00 (4.00, 6.00)	–	–	–	–
	14 weeks	5.00 (4.00, 6.00)	5.00 (4.00, 6.00)	< 0.001	0.749 (0.651–0.861)	0.094	1.156 (0.976–1.370)
	26 weeks	4.00 (3.00, 5.00)	4.00 (3.00, 5.00)	< 0.001	0.479 (0.385–0.595)	0.990	1.002 (0.739–1.359)
Group effect	*P*	–	0.932				
HR (95% CI)		–	0.974 (0.535–1.775)				
Volume of hematochezia, g/day	0 week	10.84 (9.29, 11.85)	10.70 (9.25, 11.86)	–	–	–	–
	14 weeks	9.71 (8.26, 10.79)	9.48 (8.24, 10.70)	< 0.001	0.360 (0.309–0.418)	0.236	0.869 (0.688–1.096)
	26 weeks	8.81 (7.82, 9.76)	8.78 (7.82, 9.80)	< 0.001	0.160 (0.131–0.195)	0.302	0.862 (0.650–1.143)
Group effect	*P*	–	0.763				
HR (95% CI)		–	0.913 (0.505–1.649)				

Regarding the RHI score, both groups showed significant reductions at week 26 compared to baseline. Although the combination therapy group had a slightly lower RHI score than the VDZ group, the difference between the two groups did not reach statistical significance (*P* > 0.05; Table 6).

**Table 6:** Comparison of GS and RHI improvements between the two groups.

Characteristics	Time	VDZ group (*n* = 76)	Combination group (*n* = 83)	Time effect	HR (95% CI)	Interaction effect	HR (95% CI)
				*P*		*P*	
RHI, points	0 week	16.50 (10.00, 21.00)	15.00 (9.00, 20.00)	–	–	–	–
	14 weeks	16.00 (8.25, 22.00)	14.00 (9.00, 20.00)	0.984	1.027 (0.072–14.544)	0.585	0.390 (0.013–11.428)
	26 weeks	11.00 (8.00, 14.75)	11.00 (7.00, 12.00)	< 0.001	0.018 (0.003–0.112)	0.430	0.352 (0.026–4.719)
Group effect	*P*	–	0.560				
HR (95% CI)		–	0.506 (0.051–4.982)				

The DAI scores in both groups were significantly reduced at weeks 14 and 26 of treatment compared to week 0 (*P* < 0.05). Furthermore, in the combination group, the DAI scores at both weeks 14 and 26 were significantly lower than those at week 0 and also significantly lower than those in the VDZ group (*P* < 0.05). The interaction effects also showed significant differences at 14 and 26 weeks (*P* < 0.05). Mayo ES also significantly (*P* < 0.05; Table 7). At both weeks 14 and 26 of treatment, the overall response rate in the combination group was significantly higher than that in the VDZ group (*P* < 0.05; Table 8).

**Table 7:** Comparison of treatment efficacy between the two groups.

Characteristics	Time	VDZ group (*n* = 76)	Combination group (*n* = 83)	Time effect	HR (95% CI)	Interaction effect	HR (95% CI)
				*P*		*P*	
DAI, points	0 week	247.73 (210.04, 290.91)	257.54 (215.78, 295.77)	–	–	–	–
	14 weeks	200.42 (165.83, 242.62)	172.44 (150.26, 212.33)	< 0.001	0.349 (0.050–2.422)	< 0.001	0.170 (0.027–1.061)
	26 weeks	156.07 (123.28, 186.67)	127.98 (86.69, 160.60)	< 0.001	0.599 (0.114–1.314)	< 0.001	0.752 (0.069–1.734)
Group effect	*P*	–	< 0.001				
HR (95% CI)		–	0.015 (0.002–9.158)				
Mayo ES, points	0 week	3.00 (2.00, 3.00)	3.00 (2.00, 3.00)	–	–	–	–
	14 weeks	2.00 (2.00, 3.00)	2.00 (2.00, 2.00)	< 0.001	0.692 (0.621–0.771)	0.002	0.731 (0.601–0.889)
	26 weeks	2.00 (1.00, 2.00)	2.00 (1.00, 2.00)	< 0.001	0.491 (0.421–0.573)	0.002	0.782 (0.668–0.916)
Group effect	*P*	–	0.950				
HR (95% CI)		–	1.005 (0.867–1.164)				

**Table 8:** Comparison of treatment efficacy between the two groups.

Characteristics	VDZ group (*n* = 76)	Combination group (*n* = 83)	*Z*	*P*
14-week treatment efficacy				
Overall response rate [*n* (%)]	49 (64.47)	77 (92.77)	3.138	0.002
Remission	26 (34.21)	39 (46.99)		
Effective	23 (30.26)	38 (45.78)		
Ineffective	27 (35.53)	6 (7.23)		
26-week treatment efficacy			
Overall response rate [*n* (%)]	65 (85.53)	81 (97.59)	3.355	0.001
Remission	53 (69.74)	75 (90.36)		
Effective	12 (15.79)	6 (7.23)		
Ineffective	11 (14.47)	2 (2.41)		

### Adverse reactions and safety

No statistically significant difference in adverse reactions was observed between the two groups of patients at either week 14 or 26 of treatment (*P* > 0.05; Table 9). The safety assessment indicated that, at both weeks 14 and 26 of treatment, fewer patients experienced ADRs in the combination group compared to the VDZ group. However, the OR and RR both approximated 1, suggesting that the safety profiles of the two groups were essentially comparable (Table 10).

**Table 9:** Comparison of AEs between the two groups [*n* (%)].

Characteristics	VDZ group (*n* = 76)	Combination group (*n* = 83)	χ^2^	*P*
Adverse reactions at 14 weeks			
Hepatic and renal injury			1.336	0.513
0	69 (90.79)	78 (93.98)		
1	6 (7.89)	5 (6.02)		
2	1 (1.32)	0 (0.00)		
3	0 (0.00)	0 (0.00)		
4	0 (0.00)	0 (0.00)		
5	0 (0.00)	0 (0.00)		
Infection			0.646	0.886
0	60 (78.95)	68 (81.93)		
1	9 (11.84)	10 (12.05)		
2	6 (7.89)	4 (4.82)		
3	1 (1.32)	1 (1.20)		
4	0 (0.00)	0 (0.00)		
5	0 (0.00)	0 (0.00)		
Skin lesion			1.142	0.565
0	68 (89.47)	72 (86.75)		
1	6 (7.89)	10 (12.05)		
2	2 (2.63)	1 (1.20)		
3	0 (0.00)	0 (0.00)		
4	0 (0.00)	0 (0.00)		
5	0 (0.00)	0 (0.00)		
Other AEs			1.099	0.294
0	75 (98.68)	83 (100.00)		
1	1 (1.32)	0 (0.00)		
2	0 (0.00)	0 (0.00)		
3	0 (0.00)	0 (0.00)		
4	0 (0.00)	0 (0.00)		
5	0 (0.00)	0 (0.00)		
Adverse reactions at 26 weeks			
Hepatic and renal injury			1.631	0.442
0	65 (85.53)	75 (90.36)		
1	10 (13.16)	8 (9.64)		
2	1 (1.32)	0 (0.00)		
3	0 (0.00)	0 (0.00)		
4	0 (0.00)	0 (0.00)		
5	0 (0.00)	0 (0.00)		
Infection			1.092	0.779
0	55 (72.37)	62 (74.70)		
1	11 (14.47)	14 (16.87)		
2	8 (10.53)	6 (7.23)		
3	2 (2.63)	1 (1.20)		
4	0 (0.00)	0 (0.00)		
5	0 (0.00)	0 (0.00)		
Skin lesion			1.096	0.578
0	64 (84.21)	67 (80.72)		
1	10 (13.16)	15 (18.07)		
2	2 (2.63)	1 (1.20)		
3	0 (0.00)	0 (0.00)		
4	0 (0.00)	0 (0.00)		
5	0 (0.00)	0 (0.00)		
Other AEs			2.212	0.137
0	74 (97.37)	83 (100.00)		
1	2 (2.63)	0 (0.00)		
2	0 (0.00)	0 (0.00)		
3	0 (0.00)	0 (0.00)		
4	0 (0.00)	0 (0.00)		
5	0 (0.00)	0 (0.00)		

**Table 10:** Safety profiles of the two groups.

Groups	Patients with ADRs	Patients without ADRs	Total patients	OR	RR
Safety profile at 14 weeks			
VDZ group	29	47	76	1.149	1.092
Combination group	29	54	83		
Safety profile at 26 weeks			
VDZ group	40	36	76	1.085	1.040
Combination group	42	41	83		

### Assessment of the correlation between biomarkers and DAI

**Figure 2:**
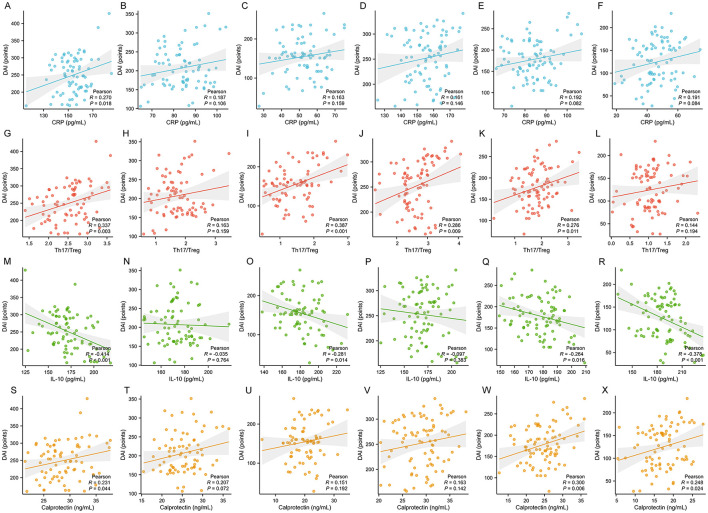
Correlation analysis between the DAI and CRP, Th17/Treg, IL-10, calprotectin. **(A, G, M, S)** VDZ group at week 0. **(B, H, N, T)** VDZ group at week 14. **(C, I, O, U)** VDZ group at week 26. **(D, J, P, V)** Combination group at week 0. **(E, K, Q, W)** Combination group at week 14. **(F, L, R, X)** Combination group at week 26.

According to the correlation analysis results presented in Figure 2, in the VDZ monotherapy group, CRP levels showed a moderate positive correlation with DAI (*P* < 0.001), the Th17/Treg ratio exhibited a weak positive correlation with DAI (*P* < 0.001), IL-10 levels demonstrated a weak negative correlation with DAI (*P* = 0.038), and fecal calprotectin levels showed a weak positive correlation with DAI (*P* < 0.001). In the combination therapy group, the correlations between these indicators and DAI were all more pronounced, with CRP showing a strong positive correlation with DAI (*P* < 0.001), the Th17/Treg ratio exhibiting a moderate positive correlation with DAI (*P* < 0.001), IL-10 demonstrating a weak negative correlation with DAI (*P* < 0.001), and fecal calprotectin showing a moderate positive correlation with DAI (*P* < 0.001).

### Subgroup results

Within the VDZ monotherapy group, the trends in immune factor levels at each time point were generally consistent between patients with moderate UC and those with severe UC. CRP, Th17/Treg ratio, IL-10, and fecal calprotectin all showed significant time effects within each subgroup (*P* < 0.05). However, intergroup comparison showed no statistically significant differences in immune factor levels between moderate and severe patients at any time point (*P* > 0.05; Table 11).

**Table 11:** Comparison of immune factors in VDZ subgroups.

Characteristics	0 week	14 weeks	26 weeks	Mauchly *W*	*P*	*F*	*P*	Partial eta squared
CRP, pg/mL								
Moderate UC (*n* = 26)	153.42 ± 11.02	82.65 ± 8.91^*^	50.23 ± 10.15^*#^	0.968	0.235	4.892	0.008	0.058
Severe UC (*n* = 50)	154.44 ± 11.58	84.56 ± 9.23^*^	53.65 ± 10.48^*#^					
*t*	−0.372	−0.864	−1.361					
*P*	0.711	0.390	0.177					
95% CI	−6.411 to 4.382	−5.480 to 2.661	−7.454 to 1.619					
Th17/Treg								
Moderate UC (*n* = 26)	2.48 ± 0.49	1.68 ± 0.55^*^	1.38 ± 0.54^*#^	0.995	0.891	8.234	< 0.001	0.096
Severe UC (*n* = 50)	2.54 ± 0.51	1.77 ± 0.58^*^	1.49 ± 0.59^*#^					
*t*	−0.498	−0.663	−0.812					
*P*	0.620	0.509	0.419					
95% CI	−0.300 to 0.182	−0.371 to 0.191	−0.392 to 0.166					
IL-10, pg/mL								
Moderate UC (*n* = 26)	170.89 ± 18.76	171.32 ± 13.98	179.28 ± 18.15^*#^	0.971	0.203	1.876	0.156	0.022
Severe UC (*n* = 50)	173.42 ± 20.12	173.94 ± 14.72	182.67 ± 19.08^*#^					
*t*	−0.538	−0.756	−0.756					
*P*	0.592	0.452	0.452					
95% CI	−12.371 to 7.312	−9.753 to 4.516	−12.149 to 5.368					
Calprotectin, ng/mL								
Moderate UC (*n* = 26)	28.21 ± 3.65	24.75 ± 4.02^*^	19.68 ± 4.58^*#^	0.993	0.812	5.234	0.005	0.051
Severe UC (*n* = 50)	29.17 ± 3.89	25.71 ± 4.28^*^	20.65 ± 4.92^*#^					
*t*	−1.060	−0.966	−0.850					
*P*	0.293	0.337	0.398					
95% CI	−2.767 to 0.859	−2.962 to 1.044	−3.257 to 1.318					

^*^P < 0.05 compared with the value at 0 week.

^#^*P* < 0.05 compared with the value at 14 weeks.

Within the combination therapy group, CRP, Th17/Treg ratio, and fecal calprotectin levels in both moderate and severe UC patients were significantly decreased at 14 and 26 weeks of treatment compared to baseline (*P* < 0.05), while IL-10 levels were significantly increased (*P* < 0.05). Intergroup comparison showed no significant differences in immune factor levels between moderate and severe patients at any time point (*P* > 0.05; Table 12).

**Table 12:** Comparison of immune factors in combination subgroups.

Characteristics	0 week	14 weeks	26 weeks	Mauchly *W*	*P*	*F*	*P*	Partial eta squared
CRP, pg/mL								
Moderate UC (*n* = 24)	153.85 ± 10.42	82.87 ± 8.31^*^	44.96 ± 10.32^*#^	0.965	0.248	5.123	0.006	0.072
Severe UC (*n* = 59)	56.73 ± 11.08	84.58 ± 8.79^*^	47.14 ± 10.98^*#^					
*t*	−1.089	−0.816	−0.835					
*P*	0.279	0.417	0.406					
95% CI	−8.094 to 2.331	−5.892 to 2.477	−6.741 to 2.375					
Th17/Treg								
Moderate UC (*n* = 24)	2.59 ± 0.51	1.78 ± 0.54^*^	1.02 ± 0.41^*#^	0.997	0.912	9.234	< 0.001	0.132
Severe UC (*n* = 59)	2.67 ± 0.54	1.86 ± 0.57^*^	1.08 ± 0.44^*#^					
*t*	−0.623	−0.590	−0.582					
*P*	0.535	0.557	0.562					
95% CI	−0.324 to 0.183	−0.355 to 0.197	−0.227 to 0.195					
IL-10, pg/mL								
Moderate UC (*n* = 24)	168.91 ± 17.85	176.23 ± 12.94^*^	185.42 ± 20.15^*#^	0.969	0.215	2.134	0.124	0.028
Severe UC (*n* = 59)	171.12 ± 18.87	178.38 ± 13.68^*^	187.94 ± 21.38^*#^					
*t*	−0.496	−0.657	−0.501					
*P*	0.621	0.513	0.617					
95% CI	−11.214 to 6.872	−8.751 to 4.416	−12.689 to 7.658					
Calprotectin, ng/mL								
Moderate UC (*n* = 24)	28.52 ± 3.92	24.21 ± 4.58^*^	16.72 ± 4.43^*#^	0.994	0.834	5.678	0.003	0.068
Severe UC (*n* = 59)	29.29 ± 4.13	24.95 ± 4.82^*^	17.40 ± 4.68^*#^					
*t*	−0.783	−0.646	−0.612					
*P*	0.436	0.520	0.542					
95% CI	−2.770 to 1.168	−2.925 to 1.474	−2.848 to 1.522					

^*^P < 0.05 compared with the value at 0 week.

^#^*P* < 0.05 compared with the value at 14 weeks.

Within the VDZ monotherapy group, DAI scores decreased significantly in both subgroups after 14 and 26 weeks of treatment (*P* < 0.05). Regarding Mayo ES, both subgroups showed significant improvement after treatment, but the difference between moderate and severe patients did not reach statistical significance (*P* > 0.05; Table 13).

**Table 13:** Comparison of treatment efficacy in VDZ subgroups.

Characteristics	Time	Moderate UC (*n* = 26)	Severe UC (*n* = 50)	Time effect	HR (95% CI)	Interaction effect	HR (95% CI)
				*P*		*P*	
DAI, points	0 week	238.45 (208.32, 278.56)	252.18 (213.45, 295.67)	–	–	–	–
	14 weeks	185.67 (158.23, 223.45)	208.34 (172.56, 252.78)	< 0.001	0.423 (0.089–2.011)	0.023	0.312 (0.089–1.094)
	26 weeks	142.34 (115.67, 172.45)	163.45 (129.87, 195.34)	< 0.001	0.487 (0.123–1.928)	0.031	0.423 (0.112–1.597)
Group effect	*P*	–	0.034				
HR (95% CI)		–	0.045 (0.005–8.234)				
Mayo ES, points	0 week	3.00 (2.00, 3.00)	3.00 (3.00, 3.00)	–	–	–	–
	14 weeks	2.00 (2.00, 2.25)	2.00 (2.00, 3.00)	< 0.001	0.712 (0.598–0.848)	0.045	0.789 (0.623–0.999)
	26 weeks	2.00 (1.00, 2.00)	2.00 (1.00, 2.00)	< 0.001	0.523 (0.412–0.664)	0.078	0.834 (0.689–1.009)
Group effect	*P*	–	0.178				
HR (95% CI)		–	0.934 (0.745–1.171)				

Within the combination therapy group, DAI scores decreased significantly in both moderate and severe patients after 14 and 26 weeks of treatment (*P* < 0.05). Regarding Mayo ES, both subgroups showed significant improvement after treatment (*P* < 0.05; Table 14).

**Table 14:** Comparison of treatment efficacy in combination subgroups.

Characteristics	Time	Moderate UC (*n* = 24)	Severe UC (*n* = 59)	Time effect	HR (95% CI)	Interaction effect	HR (95% CI)
				*P*		*P*	
DAI, points	0 week	248.67 (209.34, 285.45)	261.23 (218.56, 298.78)	–	–	–	–
	14 weeks	158.34 (138.45, 192.34)	178.56 (152.67, 218.45)	< 0.001	0.289 (0.045–1.856)	0.018	0.234 (0.056–0.978)
	26 weeks	115.45 (78.34, 145.67)	132.67 (92.34, 168.45)	< 0.001	0.478 (0.098–2.334)	0.026	0.512 (0.089–2.945)
Group effect	*P*	–	0.028				
HR (95% CI)		–	0.023 (0.003–8.456)				
Mayo ES, points	0 week	3.00 (2.00, 3.00)	3.00 (3.00, 3.00)	–	–	–	–
	14 weeks	2.00 (2.00, 2.00)	2.00 (2.00, 2.00)	< 0.001	0.689 (0.601–0.790)	0.089	0.856 (0.723–1.013)
	26 weeks	2.00 (1.00, 2.00)	2.00 (1.00, 2.00)	< 0.001	0.478 (0.389–0.587)	0.156	0.923 (0.801–1.064)
Group effect	*P*	–	0.234				
HR (95% CI)		–	0.978 (0.823–1.162)				

## Discussion

The treatment goals for moderate-to-severe UC are to maintain remission, prevent complications, and improve quality of life (8). Traditional medications for this condition are predominantly non-biological agents, which, while capable of alleviating symptoms, fail to halt the underlying inflammatory processes. VDZ reduces intestinal inflammation and alleviates symptoms by inhibiting integrins within the body, thereby preventing or mitigating further damage associated with intestinal disease progression (22). Cur may inhibit TNBS-induced ulcerative colitis by activating the PPAR-γ pathway, promoting weight recovery and reducing the macroscopic score of colitis in patients with moderate to severe UC, which may also be the main reason for the better improvement in RHI observed in this study (23).

Regarding inflammatory indicators, the combination group showed significantly greater improvement. Both ESR and PLT are sensitive markers reflecting systemic inflammatory load, and their elevation is closely associated with disease activity in UC (1). VDZ primarily acts on local intestinal inflammation by selectively inhibiting the migration of gut lymphocytes, while Cur, as a multi-target natural anti-inflammatory agent, can downregulate various pro-inflammatory cytokines, including IL-6 and TNF-α, by inhibiting key inflammatory pathways such as NF-κB (4). Therefore, the combination therapy may achieve faster and more comprehensive control of systemic inflammation through the synergistic anti-inflammatory effects of local and systemic actions, leading to significant improvements in ESR and PLT. The combination group also demonstrated significantly greater improvement in ALC and Th17/Treg ratio (9). Changes in ALC reflect adjustments in the body's immune status, while the Th17/Treg balance is a central aspect of the immune pathogenesis of UC. VDZ specifically inhibits the recruitment of effector T cells to intestinal inflammatory sites by blocking the binding of α4β7 integrin to MAdCAM-1. Cur, on the other hand, has been shown to regulate T cell differentiation, promoting immunosuppressive Treg cells while inhibiting pro-inflammatory Th17 cells (24). Therefore, combination therapy may produce synergistic effects by both blocking the homing of effector cells and regulating the balance of differentiation, thereby more effectively improving immune dysregulation in patients with UC (25). This likely explains the improvements in ALC and the optimization of the Th17/Treg ratio. Furthermore, improvements in Hb and RBC were observed in both groups after treatment, indicating that both regimens alleviated the inflammation-associated anemia commonly seen in UC (26). Although this study did not show a significant advantage for the combination group in these two indicators, Cur's known antioxidant and mucosal repair properties may help improve intestinal mucosal integrity and reduce occult blood loss, thereby supporting the long-term correction of anemia (27).

In addition to improving Th17/Treg balance, combine therapy also demonstrated favorable trends in markers such as CRP, IL-10, and fecal calprotectin, indicating that the addition of Cur may enhance the therapeutic efficacy of VDZ through multiple synergistic mechanisms (9). The significant decrease in CRP, a systemic inflammatory marker, may stem from Cur-mediated inhibition of the NF-κB pathway, which downregulates pro-inflammatory factors like IL-6 (28). This complements the localized anti-inflammatory effect of VDZ in the gut, jointly alleviating systemic inflammatory burden (2). The increase in IL-10 levels is closely linked to the ability of Cur to modulate immune balance, promoting the differentiation of anti-inflammatory Treg cells and M2 macrophage polarization, thereby fostering an anti-inflammatory microenvironment, while VDZ's control of the inflammatory source creates favorable conditions for this process (27). Interestingly, IL-10 levels were significantly higher in the combination group than in the VDZ group only at week 14, but not at week 26. This temporal pattern may reflect a more prominent role of IL-10 during the early phase of inflammation resolution, whereas its expression may plateau or be superseded by other anti-inflammatory mechanisms once disease activity is controlled (18). Moreover, IL-10 is tightly regulated by multiple immune cell subsets and signaling pathways, and its dynamic changes may parallel the restoration of Th17/Treg balance. Future studies with more frequent sampling time points are warranted to elucidate the kinetic profile of IL-10 under combination therapy (17). The notably more pronounced decline in fecal calprotectin reflects accelerated mucosal healing, benefiting from both VDZ's suppression of neutrophil recruitment to the intestine and the direct antioxidant and anti-apoptotic effects of Cur, as well as its role in repairing intestinal epithelial barrier function (29). Therefore, the advantage of the combination therapy likely lies in the precise control of gut-specific immune cell migration by VDZ, while Cur modulates systemic inflammatory responses, enhances mucosal repair, and optimizes immune balance through multi-target mechanisms, thus more effectively controlling the disease activity of ulcerative colitis across multiple dimensions (9).

In the combination group, the DAI showed a stronger positive correlation with the Th17/Treg ratio, CRP, and fecal calprotectin, while its negative correlation with IL-10 was more pronounced, indicating that the combination therapy may synergistically regulate disease activity through multiple pathways (2). The high correlation between the Th17/Treg ratio and DAI suggests that immune imbalance is a core mechanism driving the progression of UC (30). The combination therapy more effectively corrects this imbalance by inhibiting effector T-cell gut homing through VDZ while simultaneously promoting Treg differentiation and suppressing Th17 activation with Cur. The close association between CRP and DAI reflects the critical role of systemic inflammation in disease activity (30). Cur downregulates pro-inflammatory factors such as IL-6 by inhibiting the NF-κB pathway, and this effect, combined with the localized anti-inflammatory action of VDZ, collectively alleviates the systemic inflammatory burden (31). The more significant negative correlation between IL-10 and DAI in the combination group suggests that Cur may further enhance the protective role of IL-10 by improving regulatory immune cell function and promoting an anti-inflammatory microenvironment (23). The correlation between fecal calprotectin and DAI highlights the direct link between mucosal inflammation and disease activity (2). VDZ reduces neutrophil infiltration, while Cur accelerates mucosal healing and reduces calprotectin release through antioxidant, anti-apoptotic, and barrier-repair effects (16). These correlation analyses suggest that the advantage of the combination therapy may lie in its simultaneous targeting of multiple pathological processes, including immune imbalance, systemic inflammation, and mucosal damage. Through the synergistic effects of VDZ and Cur, the combination therapy more effectively controls UC disease activity across multiple dimensions (2). Although the present study did not directly measure NF-κB activity or Th17-specific cytokines such as IL-17, accumulating evidence suggests that curcumin exerts its immunomodulatory effects by inhibiting the NF-κB pathway and promoting Treg differentiation, thereby rebalancing the Th17/Treg axis (9). Thus, the observed improvements in Th17/Treg ratio and CRP levels in the combination group may be partially attributed to these molecular mechanisms. Future studies should include direct markers such as phosphorylated NF-κB or IL-17 to validate these pathways.

The results of the subgroup analysis comparing patients with moderate vs. severe UC showed that, in both the VDZ monotherapy group and the combination therapy group, the reduction in DAI scores at each time point was significantly greater in patients with moderate UC than in those with severe UC (*P* < 0.05), suggesting that patients with moderate disease derive more pronounced clinical benefit from treatment. This trend was particularly evident in the combination therapy group, indicating that the addition of Cur may further amplify the therapeutic advantage in moderate patients. The underlying reasons may be attributed to the following mechanisms. First, patients with moderate UC exhibit relatively milder intestinal barrier damage and immune dysregulation, offering greater plasticity for immunomodulatory intervention (32). Cur, by inhibiting the NF-κB pathway and downregulating pro-inflammatory cytokines such as IL-6 and TNF-α, may more effectively restore the Th17/Treg immune balance in moderate patients, thereby enhancing the localized anti-inflammatory effects of VDZ (9). Second, the systemic inflammatory burden is lower in moderate UC, with more controllable levels of systemic inflammatory markers such as ESR and CRP (26). This allows combination therapy to achieve comprehensive inflammation control more rapidly in the short term, thereby accelerating mucosal healing (23). In contrast, although patients with severe UC also showed clinical improvement with combination therapy, the degree of improvement in immune parameters and DAI was relatively limited (32). This may be associated with the more complex immune dysregulation, extensive intestinal damage, and higher inflammatory burden characteristic of severe disease (27). These findings suggest that patients with moderate UC may achieve greater net clinical benefit from Cur combined with VDZ therapy.

This study has the following limitations. First, the follow-up period was 26 weeks, while sufficient to assess mid-term efficacy and safety, is insufficient to evaluate long-term outcomes. Therefore, longer-term observation is still required in the future to comprehensively assess the long-term benefits and potential risks of the combination therapy. Additionally, although immunologic and inflammatory markers were incorporated, the study primarily relied on clinical and laboratory indicators to evaluate efficacy, lacking direct evidence at the histopathological level. Future research should involve more rigorous prospective study designs to comprehensively assess the clinical value and underlying mechanisms of this combination treatment strategy. Based on the findings of this study, future research could explore the following hypotheses. Whether the therapeutic effects of curcumin combined with VDZ are mediated through the synergistic inhibition of NF-κB and regulation of the Th17/Treg balance warrants further validation through *in vitro* experiments or intestinal tissue biopsy. Additionally, whether the early elevation of IL-10 can serve as a predictive biomarker for treatment response requires time-series analysis in larger sample cohorts.

In summary, curcumin as an combination group to VDZ significantly enhances clinical efficacy and immunomodulatory effects in patients with moderate-to-severe UC, without increasing safety risks, highlighting its important clinical translational value. This study provides new evidence for combination treatment strategies in moderate-to-severe UC, offering a foundation for optimizing therapeutic approaches and advancing the application of personalized medicine in UC management.

## Data Availability

The raw data supporting the conclusions of this article will be made available by the authors, without undue reservation.
